# Low prevalence and disease severity of COVID‐19 in post‐liver transplant recipients—A single centre experience

**DOI:** 10.1111/liv.14552

**Published:** 2020-06-17

**Authors:** Anita Verma, Shirin Elizabeth Khorsandi, Annalisa Dolcet, Andreas Prachalias, Abid Suddle, Nigel Heaton, Wayel Jassem

**Affiliations:** ^1^ Institute of Liver Studies King's College Hospital London UK

**Keywords:** COVID‐19, immunosuppression, liver transplant

## Abstract

Coronavirus disease 2019 (COVID‐19) caused by a novel coronavirus called severe acute respiratory syndrome coronavirus‐2 (SARS‐CoV‐2) is driving a present day global pandemic. Immunosuppressed patients are regarded as a high‐risk cohort. The following is a short report on COVID‐19 in liver transplant recipients (n = 5) from a high volume UK liver transplant unit with a large follow‐up cohort (n = 4500). Based on this limited data, liver transplant recipients appear to have a low incidence of COVID‐19, with less severe symptoms than expected, when compared with the general population and other solid organ recipients. This possibly could be related to self‐isolation adherence and/or the ‘ideal’ level of immunosuppression that favourably modulates the immune response to COVID‐19.

**Abbreviations**
ALF: acute liver failure
BMI: body mass index
CA: community acquired
CNI: calcineurin inhibitor
COVID‐19: coronavirus disease 2019
CPAP: continuous positive airway pressure
CXR: chest X‐ray
FAP: familial amyloid polyneuropathy
HA: hospital acquired
IS: immunosuppression
MMF: mycophenolate mofetil
PCR: polymerase chain reaction
PFIC3: primary familial intrahepatic cholestasis type 3
PHE: Public Health England
PSC: primary sclerosing cholangitis
PTC: percutaneous transhepatic cholangiogram
RNA: ribonucleic acid
SARS‐CoV‐2: severe acute respiratory syndrome coronavirus‐2
T2DM: type 2 diabetes mellitus
UC: ulcerative colitis
US: ultrasound
VOD: veno‐occlusive disease
WHO: World Health Organization

**Key points**

Liver transplant recipients appear to have a low incidence of COVID‐19, with less severe symptoms, when compared with the general population and other solid organ recipients.

## 1 INTRODUCTION

Coronavirus disease 2019 (COVID‐19), a novel severe acute respiratory syndrome coronavirus 2 (SARS‐CoV‐2), first identified in December 2019, was declared a global pandemic by the World Health Organization in March 2020. The present pandemic has spread quickly, with an exponential growth in new cases and a worldwide mortality rate of 2%‐10%.^1^ Measures imposed in many countries, including the UK have helped to reduce the number of new patients for health systems to cope.^2^

Managing patients with COVID‐19 is currently a major challenge in many countries, as the pathophysiology of COVID‐19 is not fully understood and the treatment, is mainly supportive as evidence for specific therapies is yet to be established.^3^ The host immune response as determined by the innate and adaptive systems appears to be both the determinant of COVID‐19 resolution, as well as the severity of the inflammatory response that results in SARS and death.^2^, ^4^ As a consequence, immunocompromised individuals have been classified as a COVID‐19 high‐risk group. However, emerging data suggest that liver transplant recipients (adult and paediatric) may not necessarily be at increased risk, leading to the speculation that immunosuppression (IS) may paradoxically play a protective role, by modulating the immune host response to COVID‐19.^5^

The present report is a summary of our experience of COVID‐19 in post‐liver transplant recipients.

## 2 CASES

Our Unit follows up nearly 4500 post‐liver transplant patients, of which 25% were transplanted as children. To date, only five adult patients have been identified as having COVID‐19 and none have died (see Tables 1 and 2 for demographic and clinical data summary). Two of these patients are awaiting re‐transplantation (patient 1 + 2). Patient 1 is a slim man of 36 years with no significant co‐morbidity who was originally transplanted for familial amyloid polyneuropathy in 2011. Development of a diffuse cholangiopathy led to him being relisted for redo liver transplant. He had been admitted for resitting of a blocked percutaneous transhepatic cholangiogram drain and during the admission developed a cough and low‐grade fever. He was found to be SARS‐CoV‐2 RNA PCR positive on Day 15 of his inpatient stay, with chest X‐ray (CXR) demonstrating some consolidation. Otherwise, he had reasonable graft function, but was lymphopenic from hypersplenism. No changes were made in his maintenance IS which was a low‐dose calcineurin inhibitor (CNI). He was managed conservatively and discharged for self isolation. Presently, he is suspended from the transplant waiting list until 2 nasopharyngeal swabs are SARS‐CoV‐2 RNA PCR negative. On the other hand, Patient 2, is an obese gentleman of 54 years, transplanted for Chronic Budd Chiari in 2004 with comorbidities of type 2 diabetes, high cholesterol and hypertension. Listed for retransplantation on the basis of small vessel veno‐occlusive disease causing recurrent ascites. In the community, he developed symptoms of cough and fever leading to an emergency admission to his local hospital. He was found to be SARS‐CoV‐2 RNA PCR positive and required non‐invasive ventilatory support in the form of continuous positive airway pressure. As part of his COVID‐19 management, his maintenance IS was changed from mycophenolate mofetil (MMF) and prednisone, to prednisone alone at a higher dose. He is clinically improving but remains inpatient and suspended from the transplant waiting list.

**TABLE 1:** Liver transplant recipients with COVID‐19 demographic, comorbidities and underlying liver disease. Data summary for individual patient (pt), including age in years (y), sex (male: M or female: F), race, body mass index (BMI), date of liver transplant (LT), indication for primary LT and for redo or for relisting

Pt	Age (y)	Sex	Race	BMI	Date LT (s)	Indication LT/relisting/redoLT	Comorbidity	In‐patient stay (d)	Admission reason
1^a^	36	M	Cypriot	18	2011	Familial amyloid polyneuropathy/cholangiopathy	Nil	25	Blocked PTC
2^a^	54	M	British	30.9	2004	Chronic Budd Chiari/small vessel VOD, ascites	T2DM, hypertension, high cholesterol	21	Cough, pyrexia
3	54	M	British	27.9	2009 2014	Primary sclerosing cholangitis/primary sclerosing cholangitis	UC + ileostomy	4	Headache, vomiting, photophobia, pyrexia
4	23	M	British	24.4	2017 24/1/19 20/10/19	Autosomal recessive polycystic kidney disease and congenital hepatic fibrosis/ductopenic rejection/antibody‐mediated rejection	Nil	7	Chest wall pain and fever
5	28	M	British Indian	21.4	1998 20/2/20	PFIC3/ALF	Nil	75	ALF second to variceal bleed

Abbreviations: ALF, acute liver failure; PFIC3, primary familial intra‐hepatic cholestasis type 3; PTC, percutaneous transhepatic cholangiogram; T2DM, type 2 diabetes mellitus; UC, ulcerative colitis; VOD, veno‐occlusive disease.

^a^ Marks patients who are relisted.

**TABLE 2:** COVID‐19 presentation, findings and outcome in liver transplant recipients. Summary of clinical data for each individual case

Pt	CA or HA	COVID 19 symptoms	Chest X‐ray	COVID 19 management	Other infections	WBC ×10^9^/L	Lymphocyte/neutrophil ×10^9^/L	IS regimen	IS trough Tac μg/L MMF mg/L	Liver function tests Alb g/L Bil umol/L INR Ratio AST IU/L	GFR mL/min
1	HA	Low‐grade fever, cough	Left basal consolidation	Isolation No change in IS Discharged	No	0.66	0.26/0.33	CNI	Tac 3.5	Alb 30 Bil 57 INR1.47 AST 64	>90
2	CA	Cough, fever	Consolidation RLL + ML	ABs, CPAP 3/7, MMF stopped, pred dose doubled Inpatient	No	3	NA	MMF + Pred	MMF 0.6	Alb 25 Bil 25 INR 1 AST NA	66
3	CA	No respiratory symptoms	Nil	Isolation No change IS Discharged	No	10.27	0.8/3.88	CNI + Pred Azathoprine	Tac 2.8	Alb 24 Bil 6 INR 1.17 AST 30	59
4	CA	No respiratory symptoms	Nil	Isolation No change in IS Discharged	CMV	5.22	0.66/3.46	CNI + Pred (Alemtuzumab induction 3rd transplant)	Tac 3.2	Alb 42 Bil 7 INR 1.07 AST 17	13
5	HA	No respiratory symptoms	Ground glass changes (longstanding)	No change in IS, started on remdesivir Inpatient	CMV, pulmonary IA	1.78	0.71/0.86	CNI + Pred	Tac 6.6	Alb 27 Bil 9 INR 1.03 AST 16	63

Abbreviations: ABs, antibiotics; Alb, albumin, normal range 35‐50 g/L; AST, aspartate aminotransferase, normal range 10‐50 IU/L; Bil, bilirubin, normal range 3‐20 μmol/L; CA, community acquired; CMV, cytomegalovirus; CNI, calcineurin inhibitor; CPAP, continuous positive airway pressure; GFR, glomerular filtration rate; GGT, gamma glutamyl transferase, normal range 1‐55 IU/L; HA, hospital acquired; IA, invasive aspergillosis; INR, international normalized ratio, normal range 0.9‐1.2 ratio; IS, immunosuppression; ML, middle lobe; MMF, mycophenolate mofetil; NA, not available; pred, prednisone; RLL, right lower lobe; tac, tacrolimus; WBC, white blood cell, normal range 4‐11 × 10^9^/L, lymphocyte normal range 1.3‐4 × 10^9^/L, neutrophil normal range 2.2‐6.3 × 10^9^/L.

In the remaining patients, three did not have typical COVID respiratory symptoms. Patient 3 (54 years, male) was 6 years post his second transplant for primary sclerosing cholangitis (PSC). He had no significant comorbidity apart from a high body mass index (BMI) and a longstanding end ileostomy for management of his ulcerative colitis. He presented with headache, vomiting, photophobia and fever, leading to the admitting differential diagnosis of meningitis vs COVID‐19. Lumbar puncture and CT head were normal, CXR was clear, but he was found to be SARS‐CoV‐2 RNA PCR positive. He was discharged after 3 days to continue with self isolation and no changes were made in his maintenance IS of CNI, azathioprine and prednisone. In Patient 4 (23 years, male), the presenting symptoms were of chest wall pain and fever. Their background history was of autosomal recessive polycystic kidney disease and congenital hepatic fibrosis requiring combined liver kidney transplant in 2017, then redo liver transplant in January 2019 for ductopenic rejection and October 2019 for antibody mediated rejection. For his third transplant he received Alemtuzumab induction. On admission, he had ultrasound drainage of a subphrenic collection and excision of a chest wall lesion that grew *Rhizopus oryzae* and *Cryptococcus diffluens*, so was treated with antifungals. In addition, his ECG had characteristic features for pericarditis. Initial nasopharyngeal swabs for SARS‐CoV‐2 RNA PCR were negative but on repeat, after a 7‐day inpatient stay became positive. He clinically improved and had no respiratory concerns, so was discharged home, with no change in his maintenance IS of a CNI and prednisone. In contrast, Patient 5 (28 years, male) was an inpatient when found to be SARS‐CoV‐2 RNA PCR positive. He had originally been transplanted as a child for primary familial intrahepatic cholestasis type 3 and required emergency retransplantation (February 2020) following a catastrophic bleed that induced acute liver failure in an already failing graft. He was recovering from a critical illness myopathy and was on dual antifungals for pulmonary aspergillosis. A date for discharge was planned but he started to complain of chills and throat pain, leading to COVID‐19 testing that was found to be positive on Day 67 of his admission. In view of his history and ongoing treatment for aspergillosis, he was recruited into a COVID trial and randomized to receive remdesivir (200 mg IV day 1 followed by 100 mg day 2‐5). His IS remains CNI based with the dose presently being titrated according to trough. He currently remains an inpatient, but is clinically well.

## 3 DISCUSSION

Since the outbreak of the coronavirus pandemic, the apparent incidence of COVID‐19 among our post‐liver transplant patients has been relatively low at 0.1% in comparison to the general population of 0.3% in the UK and 0.4% in the USA. All of our confirmed cases have presented with mild symptoms that have improved with routine supportive therapy, none have required invasive ventilatory support and none have died. All are adult males and severity of symptoms associated with a comorbidity profile of obesity and metabolic syndrome (hypertension, hypercholesterolaemia and diabetes). Reflecting the reported demographics of the typical COVID‐19 patient, their risk of death, with apparent sparing of children.^1^, ^3^, ^6^, ^7^ In contrast, COVID‐19 death rates post‐kidney transplant are widely reported to be >25%.

The fact that transplant recipients in the UK have been classified as very high risk (also called extremely vulnerable) for COVID‐19 by Public Health England (PHE) and have been in strict self‐isolation from the start of the UK lock down that started on the 23 March 2020 could on its own explain the reduced incidence of COVID‐19. Similar, observations and comments have come from both Italy and Spain.^5^ In the UK, testing for COVID‐19 is currently offered to symptomatic patients or recipients about to be discharged into the community (PHE guidelines version 32 updated 23 April 2020). Therefore, the true number of positive, asymptomatic recipients presently remains unknown and could be higher than the general population, if IS therapy is truly playing a ‘protective’ role in attenuating COVID‐19 symptoms. Of note, liver transplant recipients and other solid organ recipients with COVID‐19‐related symptoms in the UK have a low threshold to ensuring that their implanting unit is aware of their clinical status. So, in terms of ‘symptomatic’ COVID‐19 in the present liver transplant cohort, whether that be from the community or inpatient in their local hospital, we believe the data, despite its limitations, to be representative.

Most of the recipients in the present series presented with mild symptoms, requiring minimal supportive management and limited changes in their maintenance IS. These mild symptoms at presentation could be related to IS modulating the immune response against COVID‐19 in a beneficial manner. Reports by Carbajo‐Lozoya 2012 and Tanaka 2013^8^, ^9^ have demonstrated that CNIs such as cyclosporine and tacrolimus can reduce the viral load by inhibiting viral replication though the suppression of immunophilin pathways. Although, these observations are yet to be confirmed in a clinical setting, it is possible to hypothesize that CNIs by reducing the viral load are able to reduce the systemic inflammatory response and subsequent development of florid SARS.

There is also emerging evidence that the severe forms of SARS resulting in death are associated with an enhanced inflammatory response and cytokine storm.^10^ As a consequence, a number of clinical trials initiated for COVID‐19 are targeting the immune response, such as tocilizumab, a monoclonal antibody directed against interleukin‐6 to reduce cytokine levels. In a pilot study on 21 Chinese patients with severe COVID‐19 pneumonia, tocilizumab was reported to improve lung function^11^ and has also been demonstrated to be of benefit, in a renal transplant patient with COVID‐19.^12^ Ongoing randomized control trials will substantiate these observations. None the less, these preliminary observations are supportive of the view that IS may have a beneficial role in COVID‐19 management, rather than per se increase the risk of COVID‐19 pneumonia and death.^5^, ^13^ However, the reported COVID‐19 deaths in post‐liver transplant patients to date (n = 3), had low levels of IS but all had underlying comorbidities.^13^

In order to understand the role of IS/immune modulation in the context of COVID‐19 management, more good quality data adjusted for established COVID‐19 risk factors (eg BMI, age, gender, ethnicity, co‐morbidity) from the different solid organ recipient groups is needed. By undertaking such an analysis, will establish whether high levels of IS combined with lymphocyte depletion, is allowing a high viral load to become the main driver of the inflammatory response. Thereby, explaining the observation that renal transplant recipients appear more vulnerable to COVID‐19 than liver transplant recipients.^14^

Fortunately, in the present series, the SARS‐CoV‐2 RNA PCR‐positive liver transplant recipients have had in the most part mild symptoms that have not needed aggressive changes in management. For patients with mild self‐limiting symptoms we have not made any changes in IS. However, if there is clinical concern, our IS strategy is withdrawal of anti‐proliferative agents (ie azathioprine, MMF) and to maintain low levels of CNIs and if need be, increase the dose of steroids. Otherwise, standard of care is as for other COVID‐19 patients, that is supportive or a given therapy within a context of a clinical trial.

In general, patients with COVID‐19 present with symptoms of viral pneumonia such as dry cough, fatigue, headache and high temperature that in the second week of infection can rapidly progress to respiratory failure.^3^ But, COVID‐19 like other viral infections can underlie atypical presentations which are of note in the immunosuppressed.^15^ In the present series, a number of the liver transplant recipients had atypical symptoms, such as photophobia, headache and chest pain which is in contrast to previous reports.^5^, ^13^ Atypical presentations of COVID‐19 have also been reported in elderly frail patients and in other solid organ transplant recipients.^15^, ^16^ Highlighting, that in the present era constant vigilance is needed to minimize the spread of COVID‐19 in the healthcare environment between patients and healthcare providers.

In conclusion, liver transplant recipients appear to have a low incidence of COVID‐19, with less severe symptoms than expected compared to the general population and other solid organ recipients, possibly related to self‐isolation adherence and/or level of immune response suppression to COVID‐19.

## CONFLICT OF INTEREST

None of the authors have any conflict of interest to declare.
